# R-Spondin Potentiates Wnt/β-Catenin Signaling through Orphan Receptors LGR4 and LGR5

**DOI:** 10.1371/journal.pone.0040976

**Published:** 2012-07-16

**Authors:** Heinz Ruffner, Joëlle Sprunger, Olga Charlat, Juliet Leighton-Davies, Bianka Grosshans, Adrian Salathe, Svenja Zietzling, Valérie Beck, Maxime Therier, Andrea Isken, Yang Xie, Yue Zhang, Huaixiang Hao, Xiaoying Shi, Dong Liu, Qinhui Song, Ieuan Clay, Gabriele Hintzen, Jan Tchorz, Laure C. Bouchez, Gregory Michaud, Peter Finan, Vic E. Myer, Tewis Bouwmeester, Jeff Porter, Marc Hild, Fred Bassilana, Christian N. Parker, Feng Cong

**Affiliations:** 1 Developmental and Molecular Pathways, Novartis Institutes for Biomedical Research, Novartis Pharma AG, Postfach, Basel, Switzerland; 2 Developmental and Molecular Pathways, Novartis Institutes for Biomedical Research, Cambridge, Massachusetts, United States of America; Center for Regenerative Therapies, Germany

## Abstract

The Wnt/β-catenin signaling pathbway controls many important biological processes. R-Spondin (RSPO) proteins are a family of secreted molecules that strongly potentiate Wnt/β-catenin signaling, however, the molecular mechanism of RSPO action is not yet fully understood. We performed an unbiased siRNA screen to identify molecules specifically required for RSPO, but not Wnt, induced β-catenin signaling. From this screen, we identified LGR4, then an orphan G protein-coupled receptor (GPCR), as the cognate receptor of RSPO. Depletion of LGR4 completely abolished RSPO-induced β-catenin signaling. The loss of LGR4 could be compensated by overexpression of LGR5, suggesting that LGR4 and LGR5 are functional homologs. We further demonstrated that RSPO binds to the extracellular domain of LGR4 and LGR5, and that overexpression of LGR4 strongly sensitizes cells to RSPO-activated β-catenin signaling. Supporting the physiological significance of RSPO-LGR4 interaction, Lgr4−/− crypt cultures failed to grow in RSPO-containing intestinal crypt culture medium. No coupling between LGR4 and heterotrimeric G proteins could be detected in RSPO-treated cells, suggesting that LGR4 mediates RSPO signaling through a novel mechanism. Identification of LGR4 and its relative LGR5, an adult stem cell marker, as the receptors of RSPO will facilitate the further characterization of these receptor/ligand pairs in regenerative medicine applications.

## Introduction

The evolutionary conserved Wnt/β-catenin signaling pathway regulates diverse biological processes during embryonic development and adult tissue homeostasis. Defects in Wnt signaling have been linked to many diseases such as cancer, bone disorders, diabetes and neurodegenerative diseases [1]. The main output of Wnt signaling is to regulate the stability of β-catenin. In the absence of Wnt, β-catenin is associated with the multiprotein β-catenin destruction complex that consists of Axin, adenomatous polyposis coli (APC), and glycogen synthase kinase 3 (GSK3). In this complex, β-catenin is constitutively phosphorylated by GSK3 which triggers the binding by beta-transducin repeat containing protein (β-TrCP) and subsequent degradation through the ubiquitin-proteasome pathway. The Wnt signal is received by Frizzled and the low-density lipoprotein receptor related protein 5/6 (LRP5/6). Wnt binding induces phosphorylation of LRP5/6, and phosphorylated LRP5/6 binds to Axin, which leads to the dissociation of the β-catenin destruction complex. Stabilized β-catenin enters the nucleus, binds to the TCF transcription factors and initiates transcription of Wnt responsive genes [1], [2].

RSPO proteins are a family of secreted molecules that strongly potentiate Wnt/β-catenin signaling. There are four members of the RSPO family of proteins in vertebrates (RSPO1-4), and all four RSPO proteins stimulate Wnt signaling [3]. *Xenopus* RSPO2 was identified through cDNA expression cloning for its ability to activate the β-catenin/TCF reporter [4]. Mouse RSPO1 was shown to stimulate the proliferation of intestinal epithelia cells upon overexpression in a transgenic mouse model [5]. In both mice and *Xenopus*, RSPO proteins are often coexpressed with Wnt proteins and their expression is often activated by Wnt [4], [6], [7]. These observations suggest that RSPO may promote Wnt signaling by a positive feedback loop.

RSPO proteins contain two N-terminal furin-like, cysteine-rich domains, followed by a C-terminal thrombospondin domain. It has been shown that the furin-like domains are required and sufficient for activation of Wnt/β-catenin signaling [4]. RSPO proteins function as stem cell growth factors and thus have therapeutic potential in regenerative medicine [5], [8], [9]. RSPO1 is able to protect mice from chemotherapy-induced intestinal and oral mucositis in mice [10]. Despite the biological and therapeutic significance of RSPO the exact mechanism of RSPO-induced β-catenin activation is not well understood. It has been postulated that RSPO binds to Kremen and blocks DKK1-induced internalization of LRP6 [11]. However, this observation appears insufficient to explain RSPO action, as Kremen1/2-double homozygous mutant mice are viable and Kremen1/2-deficient fibroblasts still respond to RSPO [12]. It has also been suggested that RSPO binds to Frizzled [7] but this binding event could not be verified [4], [13]. Furthermore, binding between RSPO and LRP6 has been reported [7], [13], but this binding is also controversial [4], [11]. Since Frizzled and LRP6 are required for Wnt/β-catenin signaling, it is difficult to determine the functional significance of their interaction with RSPO towards β-catenin signaling.

We aimed to identify the RSPO receptor that mediates RSPO-induced Wnt/β-catenin signaling. Through an siRNA screen, we have identified the orphan GPCR Leucine-rich repeat containing G protein-coupled receptor 4 (LGR4) as a mediator of RSPO1 signaling. LGR4 together with LGR5 and LGR6 form a subfamily of closely related receptors. LGR proteins contain leucine-rich repeat (LRR) domains in their ectodomain which are predicted to form horseshoe like structures and serve as a possible ligand binding site [14]. The natural ligands of LGR4, LGR5 and LGR6 are unknown. LGR5 has been identified as a β-catenin target gene and it serves as a marker of resident stem cells in multiple adult tissues, such as intestine and stomach. LGR6 has been described as a skin stem cell marker [15]–[17]. Lgr4 knock-out (KO) mice show defects in bone, kidney, hair follicle, ureteric bud, gall bladder, gut and male reproductive tract development as well as impaired erythropoiesis at midgestation [18]–[25]. The results presented in this report suggest that RSPO binds to LGR4 and mediates RSPO-induced β-catenin signaling through a heterotrimeric G protein-independent mechanism.

## Results

### Loss of LGR4 Abrogates RSPO-Induced STF Activation

We used HEK293 or HEK293T cells as an *in vitro* model system to study RSPO signaling. RSPO does not activate Wnt signaling by itself, and its activity on β-catenin signaling in HEK293 cells is critically dependent on the presence of endogenous Wnt proteins [11]. It has been shown that HEK293T cells express WNT3A, and depletion of WNT3A blocked the activity of RSPO [11]. We established a HEK293T cell line stably expressing SuperTOP-Flash (STF), a β-catenin/TCF luciferase reporter construct. We postulated that RSPO functions through an unknown receptor and this receptor is required for RSPO- but not Wnt-induced β-catenin activation. To identify this putative RSPO receptor, we performed an unbiased siRNA screen using HEK293T-STF cells treated with RSPO2. siRNAs that inhibited RSPO2-induced STF activity were selected and further tested for their effect on Wnt3a-induced STF activity. Only siRNAs that specifically inhibited RSPO-induced STF activity were selected for follow-up studies. The success of this counter-screen is dependent on the lack of endogenous RSPO protein expression in HEK293T cells. Otherwise, depletion of the RSPO receptor would decrease both RSPO1- and Wnt3a-induced STF activities. Indeed, the expression of all four RSPO proteins (RSPO1-4) is minimal in HEK293T cells with C_t_ (threshold cycle) values over 30 as assessed in qPCR assays (data not shown).

**Figure 1:**
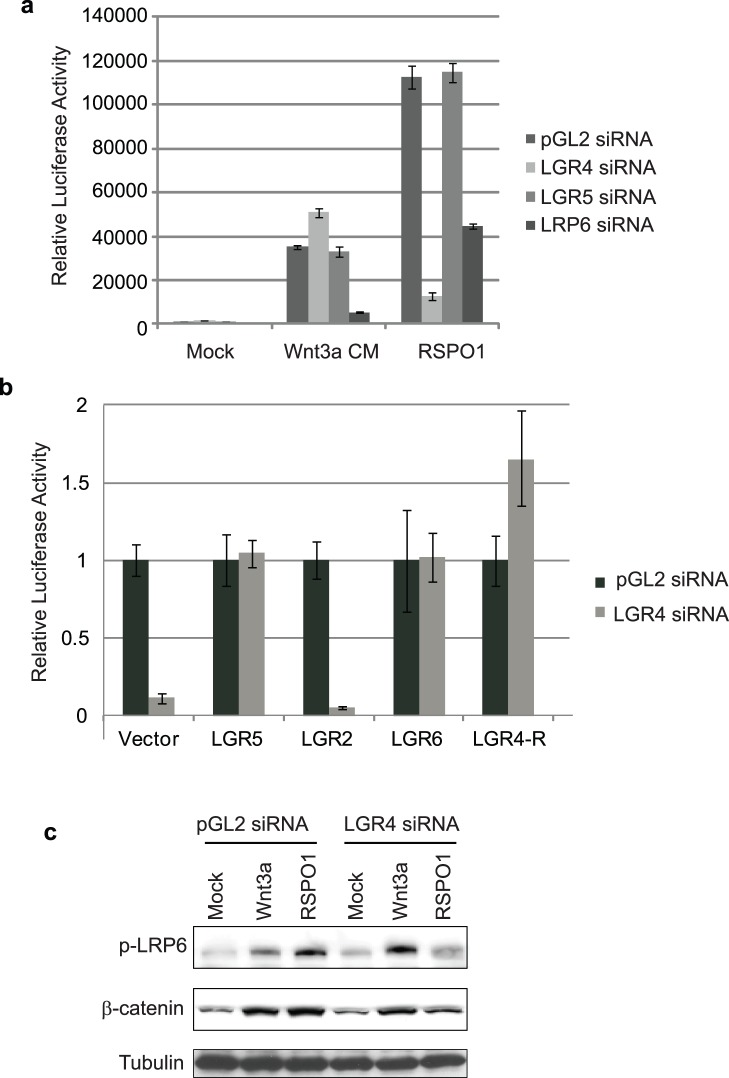
LGR4 is required for RSPO1-induced Wnt/β-catenin signaling. (a) Depletion of LGR4 inhibits RSPO1-, but not Wnt3a-, induced STF reporter activation. HEK293T-STF cells were transfected with pGL2, LGR4, LGR5, or LRP6 siRNA’s. 40 h after transfection, cells were treated with RSPO1 or Wnt3a-conditioned medium overnight, and luciferase was measured. pGL2 siRNA was used as negative control. (b) Expression of LGR4, LGR5 and LGR6 rescue LGR4 siRNA-elicited decrease in RSPO1-induced STF reporter activation. HEK293T cells stably expressing siRNA-resistant LGR4 (LGR4-R), LGR5, LGR6, LGR2 or vector control were transfected with pGL2 siRNA or LGR4 siRNA, and corresponding STF values were determined. Expression of LGR4-R, LGR5 and LGR6, but not LGR2, could rescue LGR4 loss. Luciferase values were normalized relative to pGL2 siRNA. (c) LGR4 siRNA specifically reduces RSPO1-induced LRP6 phosphorylation and β-catenin stabilization. HEK293T cells transfected with indicated siRNA’s were treated with RSPO1 or Wnt3a-conditioned medium overnight. Phospho-LRP6, cytosolic β-catenin and tubulin were analyzed by immunoblotting using indicated antibodies.

Using this screening strategy, we identified LGR4 as the only hit. As seen in Figure 1a, depletion of LGR4 strongly inhibited RSPO1-induced STF activity without affecting Wnt3a-induced activation. Several independent LGR4 siRNAs showed similar activities (Figure S1a,b; data not shown). As a control, LRP6 siRNA inhibited both RSPO1- and Wnt3a-induced STF activity. To rule out aberrant activities due to potential off-target effects of LGR4 siRNAs, we performed cDNA rescue experiments. HEK293T-STF cells stably expressing a vector control, a GFP control protein (data not shown) or a siRNA-resistant LGR4 construct were generated and transiently transfected with the pGL2 control or LGR4-specific siRNAs. As seen in Figure 1b, ectopic expression of siRNA-resistant LGR4 (LGR4-R) completely rescued the inhibitory effect of LGR4 siRNA on STF activity, suggesting that the effect of LGR4 siRNA is on-target. As a control, we showed that ectopic expression of LGR2 (also known as LHCGR, luteinizing hormone choriogonadotropin receptor, or LHR), another member of the LGR family of GPCRs [26], failed to rescue the LGR4 siRNA effect (Figure 1b). Interestingly, ectopic expression of LGR5 and LGR6 also significantly rescued the loss in STF activation induced by LGR4 siRNA (Figure 1b), suggesting that LGR5 and LGR6 are functional homologs of LGR4. We have also found that depletion of endogenous LGR5 or LGR6 had no effect on RSPO1-induced STF activation in HEK293 cells (Figure 1a and data not shown). These results suggest that LGR4 is dominant over LGR5 and LGR6, which is consistent with their lower expression compared to LGR4 in HEK293T cells (Figure S1c).

We next examined the effect of LGR4 siRNA on Wnt3a- and RSPO1-induced LRP6 phosphorylation and β-catenin stabilization. As seen in Figure 1c, depletion of LGR4 blocked RSPO1- but not Wnt3a-induced LRP6 phosphorylation and β-catenin stabilization. These results further support the conclusion that LGR4 is specifically required for RSPO-induced β-catenin signaling.

**Figure 2:**
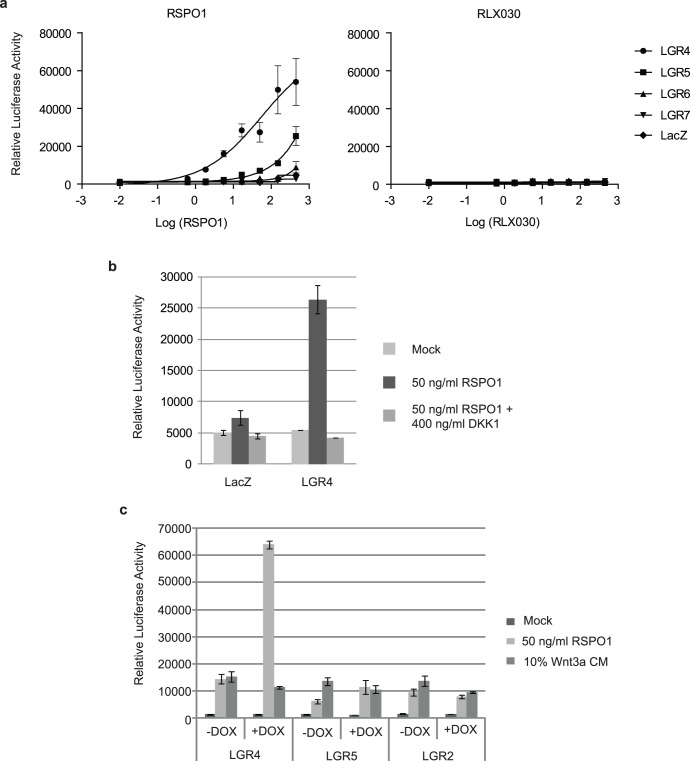
Overexpressed LGR4 homologs sensitize cells to RSPO1-induced β-catenin signaling. (a) Exogenously expressed LGR4 homologs sensitize cells to RSPO1-induced STF reporter activation. HEK293T cells were transiently co-transfected with pSTF, pRenilla and a construct encoding either LGR4, LGR5, LGR6, LGR7 or as a control LacZ. One day later, cells were treated with increasing concentrations of either RSPO1 (left; ng/ml), Relaxin 2 (RLX030, right; nM) or just medium (Mock), and luminescence values were determined 18 h later. Firefly luciferase values were normalized according to their corresponding Renilla luciferase values. Results are shown as mean +/− standard deviation of triplicate transfections. (b) DKK1 blocks LGR4-RSPO-induced Wnt pathway activation. DKK1 efficiently blocked LGR4-RSPO1-induced Wnt reporter activation. In the LacZ control transfection, the mild stimulatory effect by RSPO1 via presumably endogenous LGR4 could similarly be reversed by DKK1. (c) Synergistic Wnt pathway activation by inducible LGR4 expression and low levels of RSPO1. Stable cell lines expressing LGR4, LGR5 or LGR2 upon doxycycline treatment were stimulated with 50 ng/ml RSPO1 or 10% Wnt3a-conditioned medium (CM). LGR4 and, to a much lesser extend, LGR5 expression sensitized the cells to RSPO1-, but not Wnt3a-induced STF activation, whereas LGR2 failed to do so.

### LGR4 Overexpression Sensitizes to RSPO Signaling

We then tested whether overexpression of LGR4 can sensitize cells to RSPO signaling. HEK293T cells were transiently co-transfected with the STF reporter construct, a Renilla-luciferase construct for normalization and a plasmid encoding LGR4, LGR5, LGR6, the related receptor LGR7 or a LacZ control protein. Western blot analysis confirmed that all proteins were expressed and migrated at the expected positions on the gel (data not shown). Overexpressed LGR4 predominantly localizes to the plasma membrane, whereas overexpressed LGR5 mainly localizes to intracellular structures, as assessed for both LGR proteins in several cell types (Figure S2c and data not shown); similarly, overexpressed LGR6 is mainly localized in intracellular structures in HEK293 cells (data not shown). Cells were subsequently treated with increasing concentrations of either RSPO1 or Relaxin 2 (RLX030), the ligand of LGR7, prior to luciferase measurement after 18 h. Whereas LacZ- and LGR7-expressing cells showed only minor RSPO1-induced STF activation, presumably due to the presence of endogenous LGR4, the LGR4 overexpressing cells showed a much more robust STF induction (Figure 2a). LGR5 overexpression elicited a robust response only at high RSPO1 concentrations, whereas LGR6 revealed only a minor effect in this context. The same results were obtained when mouse Lgr4 and Lgr5 proteins were expressed in HEK293T cells and/or when mouse Rspo1 was used for stimulation, revealing conservation of the RSPO-LGR4 signaling mechanism in human and mouse (data not shown). RLX030 showed no effect on STF reporter activity upon overexpression of either receptor, even in cells transfected with its cognate receptor LGR7 (Figure 2a). Moreover, co-stimulation of overexpressed LGR4, LGR5, LGR7 and LacZ with 0.01−100 nM RLX030 and 50 ng/ml RSPO1 yielded the same STF activity values compared to RSPO1 stimulation alone, substantiating the specificity of LGR4-RSPO on Wnt signaling (data not shown). Differences in the relative plasma membrane localizations of LGR4, LGR5 and LGR6 could explain their different abilities to mediate RSPO1-induced STF activation. Alternatively, different binding affinities of these receptors towards RSPO1 could be responsible for the observed differences. Of note, the extracellular domains of LGR4 and LGR5 contain each 17 LRR repeats and display 53.4% sequence identity, whereas the extracellular domain of LGR6 contains only 13 LRR repeats. We also found that the activity of RSPO1-induced STF activation in LGR4 overexpressing cells is completely blocked by recombinant DKK1 (Figure 2b), consistent with the notion that RSPO requires endogenous Wnt proteins for its activity. Furthermore, HEK293T-STF cells that inducibly express GFP, LGR4, LGR5, and LGR2 were generated and treated with different doses of RSPO1 or Wnt3a-conditioned medium. As seen in Figure 2c, doxycycline (DOX)-induced overexpression of LGR4 significantly enhanced STF activation at low RSPO1 concentrations. However, overexpression of LGR4 did not affect Wnt3a-induced STF activation. In agreement with the above observations, overexpression of LGR5 also slightly increased STF activity at low RSPO1 concentrations, whereas overexpression of LGR2 had no effect. These results suggest that overexpression of LGR4 sensitizes cells to RSPO signaling.

### Interaction of LGR4 and RSPO1

**Figure 3:**
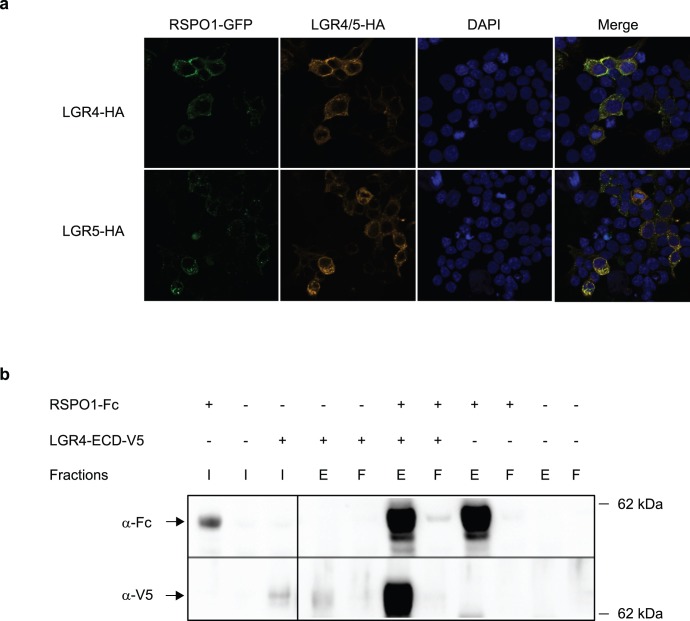
RSPO1 interacts with the extracellular domain of LGR4 and LGR5. (a) RSPO1 binds to cells overexpressing LGR4 or LGR5. HEK293T cells transiently overexpressing HA-tagged LGR4 or LGR5 were incubated with RSPO1-GFP-conditioned medium for 1 h at 37°C and subjected to immunofluorescence analysis. RSPO1-GFP only binds to cells overexpressing LGR4 or LGR5. Also note the co-localization of RSPO1-GFP and LGR4 or LGR5 in some intracellular vesicular structures. (b) Co-immunoprecipitation of LGR4-ECD and RSPO1. HEK293T cells were transiently transfected with plasmids expressing either C-terminally V5-6xHis-tagged LGR4-ECD or Fc-tagged RSPO1. Supernatants of these cultures and of control non-transfected HEK293T cells were mixed in combinations as indicated and subjected to Fc-pulldown experiments. Eluates (E), flow-through fractions (F) and input lysates (I) were analyzed by Western Blot analyses. Immunoreagents against IgG or V5 were applied to detect RSPO1 (a-Fc) and LGR4-ECD (α-V5), respectively. The position of the 62 kDa marker band is indicated on the right.

We next examined whether RSPO physically interacts with LGR4. Using a cell-based assay, HEK293T cells were transiently transfected with HA-tagged LGR4 or LGR5 and incubated with RSPO1-GFP conditioned medium. The binding of RSPO1-GFP to LGR4 or LGR5 expressing cells was evaluated by immunofluorescence microscopy. As shown in Figure 3a, RSPO1-GFP bound to cells overexpressing LGR4 but not to cells without LGR4 (data not shown). We also detected the binding of RSPO1-GFP to LGR5-expressing cells. Interestingly, RSPO1-GFP was often co-localized with LGR4 and LGR5 in intracellular vesicles, suggesting co-internalization of RSPO1-GFP with LGR4 and LGR5. To further test whether RSPO1 and LGR4 are capable of directly interacting with each other, we performed protein pulldown assays. Conditioned media containing Fc-tagged RSPO1 and V5-tagged extracellular domain (ECD) of LGR4 were mixed and incubated with Protein A beads. Aliquots of precipitates and flow-through fractions were assessed by immunoblot analysis using anti-Fc or anti-V5 immunoreagents (Figure 3b). The results demonstrate that LGR4-ECD-V5 was co-precipitated with RSPO1-Fc, implicating a direct interaction. Together these results suggest that RSPO interacts with the extracellular domain of LGR4 and LGR5.

To elucidate which part of the LGR4 protein is responsible for the RSPO1-induced Wnt pathway activation, we functionally assessed two LGR4 deletion mutants in a transient transfection experiment using HEK293T-STF cells (Figure S2a). Deleting the C-terminal cysteine (Cys)-rich domain of the ECD (ΔC-Cys) or the extreme C-terminus of the intracellular domain (ICD; ΔC-term) had little effect on RSPO1 activity, even though the former was only poorly expressed, as monitored by Western blot analysis (Figure S2b). Wild-type LGR4 and the 2 mutants were localized to the plasma membrane (Figure S2c). This finding suggests that the C-terminal Cys-rich domain of the ECD is not involved in mediating RSPO1 function. Deleting the LRR domain or the N-terminal Cys-rich domain of the ECD led to a strong decrease in RSPO1-induced STF activation; however, since these two mutants were mainly localized intracellularly, it is not clear yet whether the decrease in STF reporter activation is due to lack of interaction surface for RSPO and/or to altered sub-cellular localization of the LGR4 mutants (data not shown). Further biochemical studies will be needed to map the exact interaction surface of LGR4 and RSPO1.

### Secretomics Screen

**Figure 4:**
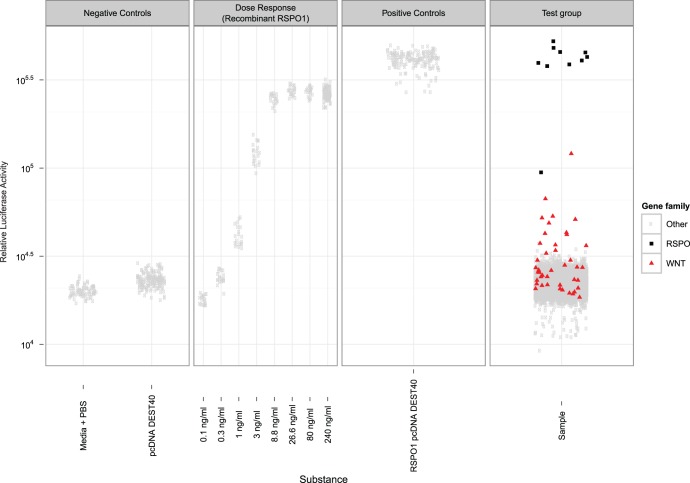
Secretomics screen on LGR4-STF HEK293 cells. A HEK293 cell line harboring stably integrated LGR4 and the STF reporter construct were incubated with conditioned media of a library of 2859 proteins known or predicted to be secreted. Firefly luciferase values were determined for each sample and normalized to Alamar blue values of the respective samples. Media + PBS and pcDNA DEST40: Negative controls, not expressing any secreted protein; recombinant RSPO1 samples, positive controls using recombinant RSPO1 at concentrations ranging from 0.1−240 ng/ml; RSPO1 pcDNA DEST40, positive control expressing RSPO1 cDNA; Sample: Combined dataset of all secreted (known or predicted) proteins, each protein assessed from conditioned media of duplicate cDNA transfections. RSPO1-4 proteins (black squares) scored highest. Then WNT proteins (red triangles) WNT1, WNT2, WNT3A, WNT7B and WNT8A reproducibly scored above a threshold of 10^4^.^5^ that marks the upper range of the bulk of datapoints, whereas WNT2B, WNT5A, WNT5B, WNT6, WNT8B, WNT9B, WNT10A, WNT10B, WNT11 and WNT16 scored below; WNT3, WNT4, WNT7A and WNT9A centered around the threshold. Each dot, triangle or square represents the luciferase value of a unicate transfected cDNA construct. Shown is an aggregate display of the primary screen and hit confirmation analysis.

*In vivo* studies have shown that Lgr4-deficient mice displayed defects associated with decreased Wnt signaling in multiple organs, such as kidney, skin, and bone [18]–[20]. Our studies suggest that RSPO proteins are the ligands of LGR4. However, it is still possible that LGR4 has other ligands which also potentiate Wnt/β-catenin signaling. To identify such factors, we performed a screen assessing the effect of secreted proteins (a “secretomics screen”) in HEK293-STF cells overexpressing LGR4. This screen used conditioned media from the complement of 3432 predicted secreted human proteins available to us for testing. From this screen, we identified all RSPO1-4 proteins as strong activators and several Wnt proteins as intermediate activators of β-catenin signaling (Figure 4). When RSPO proteins were assessed in parallel on HEK293T-STF cells not overexpressing LGR4, the amplitude of Wnt reporter activation was much weaker (data not shown), confirming our previous findings that overexpression of LGR4 potentiates RSPO signaling. However, we did not identify any other factor that effectively potentiated β-catenin signaling in this setting. These results suggest that RSPO proteins are likely the only ligands of LGR4 for β-catenin signaling.

### RSPO and Lgr4 are Essential for Small Intestine Embryo Organoid Growth

**Figure 5:**
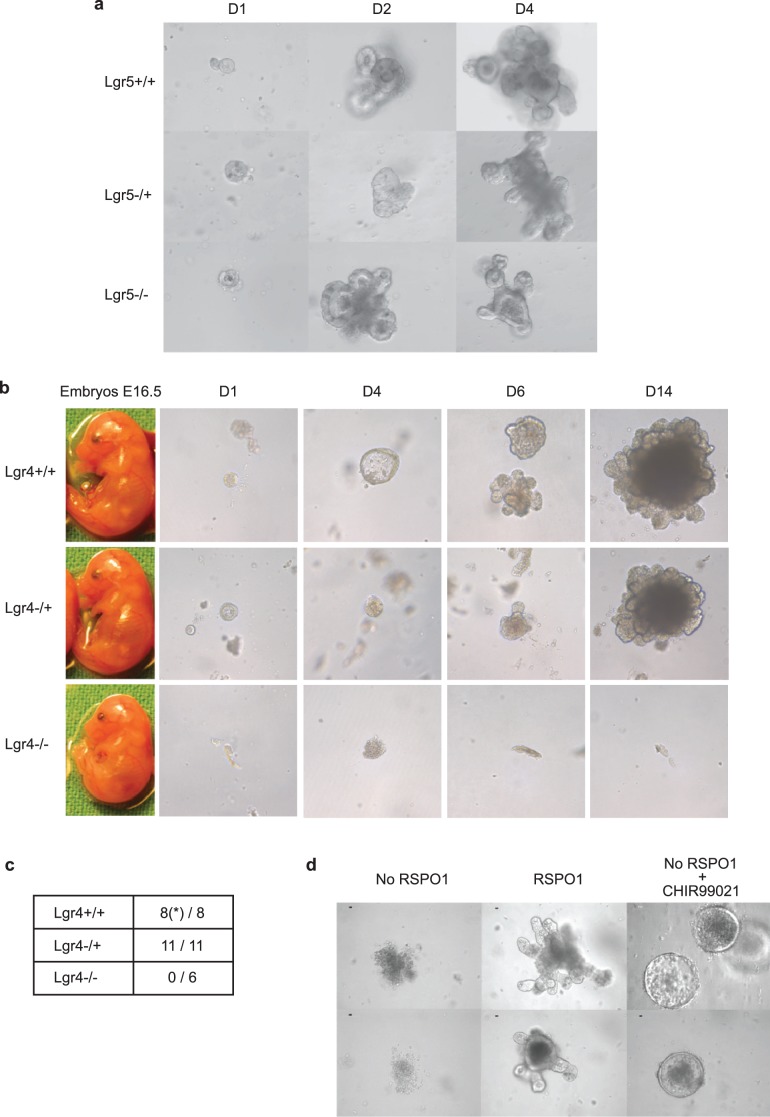
Lgr4-RSPO1-potentiated Wnt/β-catenin activation is critical for organoid survival. (a) Embryonic organoid cultures from Lgr5 (−/−), Lgr5 (−/+) and Lgr5 (+/+) are viable. Organoid cultures from E18.5 embryos of heterozygous Lgr5 KO timed matings were established. Depicted are representative organoids at day 1 (D1), day 2 (D2) and day 4 (D4) following passaging. (b) Lack of embryo Lgr4 (−/−) organoid culture growth. Organoid cultures from E16.5 embryos of heterozygous Lgr4 KO timed matings were established and allowed to grow in culture under standard growth conditions. Viability was scored by microscopic analysis over at least 2 weeks. Left column, representative E16.5 embryo of each genotype; columns 2–5, depicted are representative organoid cultures at day 1 (D1), day 4 (D4), day 6 (D6) and day 14 (D14) following passaging. Whereas wild-type (+/+) and heterozygous Lgr4 (−/+) cultures grew out, homozygous mutant Lgr4 (−/−) cultures could not be obtained. (c) The numbers shown in the table indicate the numbers of organoid cultures that grew out offset against the number of organoids prepared of each genotype; (*), one (+/+) culture grew for 1 week but subsequently lost viability for unknown reasons. (d) Lack of organoid growth upon RSPO1 withdrawal could be overcome by addition of a GSK3 inhibitor. Organoid cultures derived from adult C57Bl/6 mice were grown under standard conditions. At passage 2, multiple aliquots of sub-cultures were assessed for growth under the following conditions: No RSPO1, RSPO1 withdrawal; RSPO1, presence of 10 ng/ml RSPO1; No RSPO1+ CHIR99021, 5 uM CHIR99021 in the absence of RSPO1. Organoid growth was monitored over 8 days by microscopy. CHIR99021 was able to partially rescue organoid growth. Shown are 2 representative cultures for each condition (top and bottom, each). Note that the organoids treated with the GSK3 inhibitor reveal a cyst phenotype without crypts.

To functionally study the connection between RSPO and Lgr4 or Lgr5 *in vivo*, we made use of Lgr4 and Lgr5 KO mice. It has previously been shown that Lgr5(+) stem cells isolated from small intestine can form crypt organoid cultures *in vitro* in the presence of Paneth cells without a mesenchymal niche, and that exogenously added RSPO1 is critical for the survival of these organoids [8], [27]. Since Lgr4 and Lgr5 KO mice are both perinatally lethal [28], [29], we generated intestinal organoid cultures from E16.5 or E18.5 embryos that are either wild-type, heterozygous or homozygous for Lgr4 or Lgr5 deletions, respectively (manuscript in preparation). Whereas embryo organoid cultures of all three Lgr5 genotypes could readily be generated, we failed to obtain organoid cultures homozygous for Lgr4 deletion (Figure 5a–c). In contrast, organoid cultures from heterozygous Lgr4-deficient and wild-type embryos could be established. These data suggest that RSPO1-Lgr4-mediated activation of β-catenin signaling is critically important for organoid growth. If the Wnt pathway is the main effector of the RSPO-Lgr4 interaction in organoid cultures, downstream activation of the Wnt pathway should overcome the requirement of RSPO1-Lgr4 deficiency for organoid growth. Indeed, addition of the GSK3 inhibitor CHIR99021, which is able to activate the STF reporter by itself (data not shown), leads to organoid survival in the absence of RSPO1, most likely due to Wnt pathway activation (Figure 5d). Organoids grew as cyst-like structures without crypts, similar to the initial growth of Lgr4/5-deficient organoids treated with CHIR99021 [30].

### LGR4 Does not Appear to Signal via G Protein Activation

**Figure 6:**
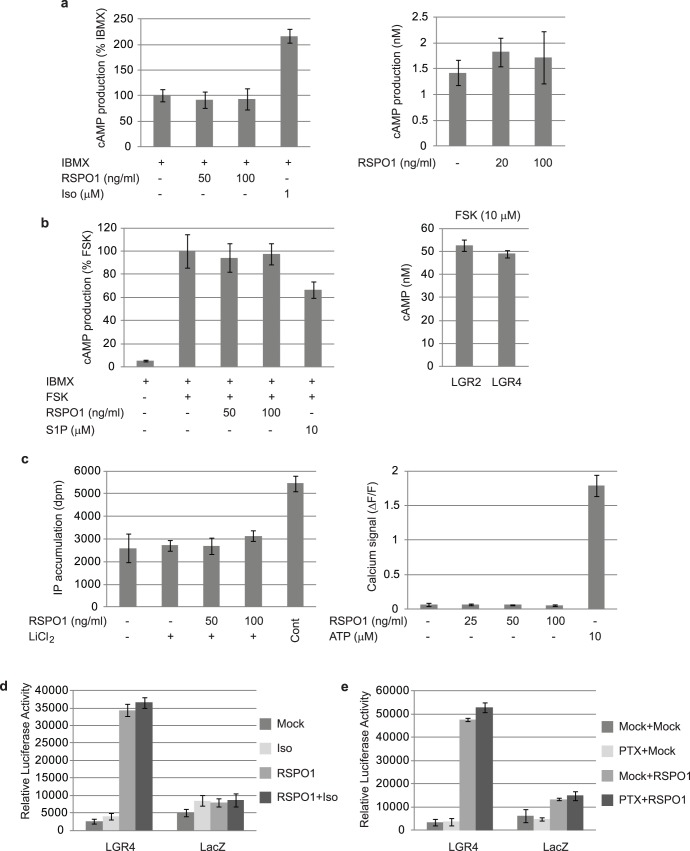
RSPO1-LGR4-induced β-catenin activation is not mediated by GPCR signaling. (a) RSPO1-LGR4 does not signal via G_αs_. (Left) HEK293 cells overexpressing LGR4 where incubated with IBMX (phosphodiesterases inhibitor) either alone or in presence of RSPO1. Isoproterenol (Iso), an agonist of the endogenously expressed β_2_-adrenergic receptor, was used as a positive control of G_s_ signaling. cAMP accumulation was determined as described in Materials and Methods. (Right) Similar as in (a), but without IBMX. (b) RSPO1-LGR4 does not signal via Gi. (Left) Forskolin (FSK, adenylyl cyclase stimulator)-stimulated cAMP levels are not reduced by RSPO1 in LGR4-overexpressing cells. Sphingosine-1-phosphate (S1P, agonist of the endogenous S1PRs) served as a positive G_i_ signaling control. cAMP accumulation was determined as described in the Materials and Methods section. (Right) LGR4 expression did not cause decreased cAMP levels when compared to LGR2 in FSK-treated cells. (c) RSPO1-LGR4 does not signal via G_αq_. HEK293 cells overexpressing LGR4 where incubated with LiCl_2_ (inositol phosphate phosphatases inhibitor) either alone or in presence of RSPO1. A compound mix (see Material and Methods) served as a G_q_ signalling positive control. Inositol phosphate accumulation was determined as described in the Materials and Methods section. Similarly, no increase in calcium release was measured upon RSPO1 treatment (right) while a positive control (ATP, agonist of the endogenous P2RYs) is activating the cells. (d) HEK293T cells were transiently transfected with pSTF, pRenilla and cDNA constructs expressing LGR4 or the LacZ control. Cells were stimulated for 24 h as follows before luciferase values were determined as described in Figure 2a: Mock, medium; Iso, 1 uM Isoproterenol; RSPO1, 50 ng/ml RSPO1; RSPO1+Iso, 50 ng/ml RSPO1+1 µM Isoproterenol. (e) The same reporter and cDNA constructs were transfected into HEK293T cells as in Figure 6d, and luciferase values were determined after the following pre-treatments and subsequent stimulations, separated by a PBS wash, each: Mock+Mock, 4 h medium pre-treatment followed by 24 h medium treatment; PTX+Mock, 4 h pre-treatement with 100 ng/ml pertussis toxin (PTX) followed by 24 h medium treatment; Mock+RSPO1, 4 h medium pre-treatment followed by 24 h 50 ng/ml RSPO1 treatment; PTX+RSPO1, 4 h pre-treatment with 100 ng/ml PTX followed by 24 h 50 ng/ml RSPO1 treatment.

Our results strongly suggest that LGR4 and LGR5 function as the receptors of RSPO. Both are predicted to be members of the GPCR superfamily, and other LGRs such as LGR1 (FSHR), LGR2 (LHR) and LGR3 (TSHR) are coupled to heterotrimeric G proteins. LGR2 for example could be activated by hCG to elicit an increase in cAMP production which is characteristic of Gs signaling, but LGR2 could not stimulate STF reporter activation in response to hCG treatment (Figure S3). LGR4-expressing cells, however, did not display the same behavior as LGR2-expressing cells. We monitored no increased cAMP levels following addition of increasing concentrations of RSPO1 in the presence of the phosphodiesterase inhibitor IBMX, showing that RSPO1 did not induce Gs through LGR4 (Figure 6a). Likewise, RSPO1 did not induce a decrease in forskolin-stimulated cAMP production in LGR4-expressing cells, indicating no Gi coupling of this receptor (Figure 6b, left). Moreover, forskolin-treated LGR4-expressing cells had the same levels of cAMP as cells expressing LGR2 (Figure 6b, right). It has been suggested that a constitutively active mutant of LGR4, T775I, is able to increase cAMP levels in a constitutive manner [31], [32]. In our hands, this mutant form of LGR4 displays the same sub-cellular localization and RSPO1-induced Wnt/β-catenin activation like wild-type LGR4. However, the T775I does not lead to elevated cAMP levels (data not shown). Activation of the Gq pathway induces an increase in inositol phosphate formation followed by calcium release from intracellular calcium stores. As demonstrated in Figure 6c, addition of RSPO1 to LGR4-expressing cells did neither increase inositol phosphate formation nor triggered calcium release while the cells responded to a positive control. This demonstrates that the Gq pathway is not involved in RSPO1-induced β-catenin/Wnt signaling. Conversely, to demonstrate that the RSPO1-LGR4-induced Wnt/β-catenin reporter activation is not due to G protein signaling we subjected LGR4-expressing HEK293 cells to isoproterenol treatment which leads to increased cAMP levels (Figure 6a). As shown in Figure 6d, isoproterenol did not affect LGR4-RSPO1-induced Wnt/β-catenin signaling. Similarly, pre-treatment of cells with pertussis toxin, which negatively interferes with Gi signaling, had no effect on LGR4-RSPO1-induced Wnt/β-catenin activation (Figure 6e). Taken together, these results suggest that LGR4-dependent RSPO1 modulation of the Wnt/β-catenin pathway does not involve G_i_, G_s_ or G_q_ protein activation.

## Discussion

Using an unbiased screening strategy, we have identified LGR4 and LGR5 as receptors of RSPO proteins. Depletion of LGR4 completely abolishes RSPO1 signaling in HEK293T cells, while overexpression of LGR4 potentiates RSPO1-4 signaling. We also showed that RSPO1 physically interacts with the extracellular domain of LGR4 and LGR5. Further, RSPO1 does not induce the coupling between LGR4 and heterotrimeric G proteins, suggesting that LGR4 transmits RSPO signaling through a novel, as of yet undefined, mechanism. During the preparation of this manuscript, three studies were published suggesting that LGR4 and LGR5 are receptors for RSPO [30], [33], [34]. Whereas one study identified physical RSPO1-LGR4/5 interaction using a hypothesis-based cell-based binding approach [33], the other two studies applied a proteome- and genome-wide strategy, respectively. The former used affinity purification-mass spectrometry-based experiments to identify binding among LGR4/5, RSPO, LRP5/6 and Frizzled proteins [30], the latter used an siRNA-based screen to identify functional LGR5-Rspo3 interaction [34]. Our results are fully consistent with these publications, and further support the conclusion that RSPO potentiates Wnt/β-catenin signaling through LGR4 and LGR5. It should be noted that we identified the RSPO-LGR4 ligand-receptor pair through complementary unbiased genome-wide screening efforts based on loss (RNAi)- and gain (complement of secreted factors)-of-function approaches. These studies suggest that RSPO proteins are the sole cognate ligands of LGR4 for canonical Wnt/β-catenin signaling.

Our findings are fully consistent with previously published *in vitro* and *in vivo* data. Rspo1 is expressed in Paneth cells which form the niche for Lgr5+ stem cells within the intestinal crypts [27]. In *ex vivo* crypt organoid cultures, RSPO is critical for the proliferation of Lgr5+ stem cells [9], [27]. Overexpression of RSPO induces massive proliferation of Lgr5+ stem cells [5], [9]. Mouse KO studies also support a role for Lgr4 in Wnt signaling. Lgr4-deficient mice have decreased bone formation, which is consistent with a critical role of Wnt signaling in bone formation [18]. Lgr4-deficient mice also showed impaired ureteric bud differentiation during kidney development, which is likely caused by down-regulation of Wnt signaling [21]. Lgr4-deficient crypts proliferate very poorly if at all in the crypt culture assay, which can be partially rescued by addition of Wnt3a [30] or LiCl [23]. Similarly, RSPO1 deficiency can be rescued by addition of a GSK3 inhibitor (our study). Lgr4 deficiency has been attributed to defective Paneth cell differentiation [23]. It remains to be seen whether this is due to a direct effect of a lack in Lgr4-RSPO signaling on Paneth cells or an indirect effect of decreased intestinal stem cell differentiation into Paneth cells. As a consequence, altered production of the Lgr4/5 niche factor RSPO1 within Paneth cells might affect stem cell activity in turn. Our study has demonstrated that organoid cultures can be generated from E16.5 embryos at a time when the morphogenesis of the mouse small intestinal epithelium is still in progress and the crypts have not yet formed [35]. Our study suggests that the cellular machinery necessary for stem cell function and crypt development must already be present at this stage, since the resulting organoid cultures, once established, are very similar in morphological appearance compared to organoid cultures generated from adult mice.

It is compelling that the RSPO-LGR4 interaction does not activate the G_i_, G_s_ or G_q_ pathways. Structurally, LGR4 and LGR5 are quite similar to other leucine-rich repeat-containing GPCRs which are coupled to heterotrimeric G protein signaling by ligand binding. It is still unclear how RSPO and LGR4 potentiate Wnt/β-catenin signaling and whether there are additional receptors for RSPO besides LGR4 and LGR5. Moreover, ligands that can induce the coupling of LGR4 and LGR5 to trimeric G proteins might still exist, although such ligands might not play a role in Wnt signaling.

From a therapeutic perspective, the RSPO-LGR4/5 angle represents an attractive opportunity for Wnt pathway agonism. Transient enforcement of the RSPO-LGR4/5 axis will lead to transiently enhanced Wnt pathway activation and might therefore be beneficial for conditions that display reduced Wnt pathway activity or that would be positively impacted by Wnt pathway activation, such as tissue or organ stem cell-driven processes like regeneration.

## Materials and Methods

### Cell Culture, Transfection and RNA Interference

HEK293T (ATCC, cat. No. CRL-11268) cells were cultured in D-MEM/F12 (Gibco #31885 and #21765, respectively), 10% FBS (Amimed #2-01F12-1) and 1% penicillin/streptomycin (Animed #4-01F00-H). Cells were cultured at 37°C, 5% CO_2_. HEK293T-STF cells were established by stably transfecting STF reporter into HEK293T cells [36]. For LGR4 and LGR5 expressing cells, geneticin (G418) was additionally added to a final concentration of 0.4mg/ml (Gibco #10131). Plasmid or siRNA transfection was done using Fugene HD (Roche) or Lipofectamine 2000 (Invitrogen). siRNA transfection was done using Dharmafect 1 (Dharmacon). Sequences of siRNAs used are listed as follows: LRP6 (Dharmacon J-003845-11), target sequence, 5′ -CCACAGAGCGAUCACAUUA-3′. LGR4-1 (Dharmacon J-003673-07), target sequence, 5′-AGGAUUCACUGUAACGUUA-3′. LGR4-2 (Dharmacon J-003673-08), target sequence, 5′-UUACUGAAGCGACGUGUUA-3′. CTNNB1, sense, 5′-UGUGGUCACCUGUGCAGCUdTdT-3′; antisense, 5′-AGCUGCACAGGUGACCACAdTdT-3′.

### STF Assay

The following DNAs were complexed with Lipofectamine 2000 (Invitrogen) following the manufacturer’s instructions, indicated as total amount per well in a 96-well plate: 20 ng pSTF, 2 ng pRenilla [36] and 58 ng pcDNA3.1 plasmid encoding the respective cDNA inserts. DNA mixes were added, in triplicate, to HEK293T cells that were plated the day before at a density of 2×10e^4^ cells per well in 96-well plates (Perkin Elmer). At 24 or 48 h following start of transfection, cells were treated with fresh medium for 24 h in the presence of absence of 50 ng/ml RSPO1 (Sino Biological Inc.), 20% Wnt3a-conditioned medium [36] or 100 nM RLX030, the recombinant form of human Relaxin 2 (Novartis Pharma AG). Firefly and Renilla luminescence signals were measured using the Dual-Glo luciferase assay system (Promega). Firefly luminescence signals were normalized according to their corresponding Renilla signals.

### Secretomics cDNA Screening Using STF Reporter Cells

cDNAs were obtained from the Ultimate *ORF* collection of secreted proteins (Invitrogen) and robotically stamped into 384-well format, 30 ng per well. Using Fugene HD (Roche), the transfection reagent and cDNAs were complexed at room temperature before HEK293T cells were added to start the reverse transfection. Cell plates were then stored at 37°C, 5% CO_2_ for 72 h.

HEK293T-LGR4-STF cells were plated into white, opaque matrix 384-well plates using standard lab automation, and were incubated at 37°C, 5% CO2 for 24 h. The secreted protein supernatant from the cDNA plate was transferred from the transfection plate (after 72 h incubation) to the assay plate and incubated for a further 16–20 h. Recombinant RSPO1 was used as a positive control, as well as pcDNA DEST40 (Invitrogen) expressing RSPO1, and a recombinant protein dose response was added to each plate to control for plate to plate variability; pcDNA DEST40 empty vector served as negative control. Steady Glo (Promega) was added to the assay plates and following a 5 min room temperature incubation, the assay was read on the Envision (Perkin Elmer) using a 0.1 s/well protocol for US luminescence giving a value of “relative light units” (RLU) for each test well.

### Solution-based and Cell-based Binding Assays

HEK293T cells were transiently transfected with either C-terminally V5-6xHis tagged LGR4-ECD or Fc-tagged RSPO1 or left untransfected for 16 h before medium change using DMEM 0.2% FBS. Supernatants of these cultures were harvested and subjected to co-immunoprecipitation experiments using the combinations. In brief, supernatants were mixed and incubated for 4 h at 4°C while tumbling. Protein A Sepharose beads (GE Healthcare) were added and incubated for 1 h at 4°C while tumbling. Immunoprecipitates were centrifuged for 2 min at 1350 g and the supernatants were removed (unbound fractions). Aliquots were secured for subsequent Western blot analyses. The immunoprecipitates were washed 5-times with 25 mM NaF, 1 mM DTT, 0.2% NP40 and once with 25 mM NaF, 1 mM DTT before 2x LDS loading buffer (Invitrogen) was added to the beads. The precipitated protein complexes were boiled off from the beads (eluates) in SDS-loading buffer and applied to SDS-PAGE and Western blot analysis using V5- or IgG-specific immunoreagents.

For cell-based binding assays, HEK293T cells were transiently transfected with plasmids encoding C-terminal HA-tagged LGR4 or LGR5. 48 h after transfection, cells were incubated with RSPO1-GFP conditioned medium for 1 h at 37°C. Cells were washed with PBS, fixed with 4% paraformaldehyde, stained with anti-HA antibody, and analyzed by fluorescence microscopy.

### Western Blot Analysis

Total cell lysates were prepared by lysis in PLCLB buffer (50 mM Hepes pH 7.4, 150 mM NaCl, 10% glycerol, 1% Triton-X100, 15 mM MgCl_2_, 10 mM EGTA, 1 mM DTT) by repeated vortexing and clearing by centrifugation. Lysates were normalized for protein concentration, resolved by SDS-PAGE, transferred onto nitrocellulose membranes and probed with the indicated antibodies. To generate cytosolic lysates, cells were scraped into hypotonic buffer (10 mM Tris-HCl pH7.5, 10 mM KCl), and cleared by centrifugation after four freeze-thaw cycles. In all experiments, 1x protease inhibitor cocktail (Roche) and 1x phosphatase inhibitor cocktail (Upstate) were added into the lysis buffers. The source of primary antibodies is as follows: anti-LRP6, anti-pS1490 LRP6 (Cell Signaling technology), anti-V5 (Invitrogen), anti-HA (Roche), anti-β-catenin (BD Pharmingen), anti-tubulin (Sigma). For detection of Fc-tagged RSPO1, protein A-HRP (Invitrogen) was used.

### Plasmids

The coding sequences of LGR2, LGR4, LGR5 and LGR7 were tagged with either HA or V5 epitopes at the C-termini and cloned into mammalian expression vectors under control of the CMV promoter. LGR4 cDNA resistant to LGR4-1 siRNA was generated by PCR-based mutagenesis. The following LGR4 mutants were generated by directed mutagenesis in the pcDNA3.1 plasmid backbone (Invitrogen): Deletion mutants encompassing amino acids 25–61, 62–464, 465–533 or 930–951 as well as point mutant T755I. All mutant plasmids were sequence-verified. RSPO1-GFP was cloned by fusing GFP to the C-terminus of RSPO1. Fc-RSPO1 was cloned by fusing mature RSPO1 to the C-terminus of the Fc tag.

### Cyclic AMP Measurements

Confluent cells grown in 48-well plates were loaded with [^3^H]Adenine (100 MBq/ml; Amersham) for 4 h in serum-free D-MEM medium (Gibco) supplemented with 1% penicillin/streptomycin (Animed). The medium was removed, cells were washed with HBSS buffer (Gibco) and subsequently supplemented with 90 µl of HBSS containing 1 mM IBMX (Fluka) in order to block phosphodiesterases. Where indicated, 90 µl of HBSS containing compounds and/or Forskolin (Sigma, final concentration 40 µM) were added per well. Cells were then incubated for 20 min at 37°C. Following incubation, the medium was removed and replaced with 90 µl of ice-cold 5% trichloroacetic acid (TCA, Fluka) and supplemented with 90 µl of ice-cold carrier solution containing 0.1 mM ATP (Boehringer) and 0.1 mM cAMP (Boehringer) in 5% TCA. Labeled cAMP was separated from free adenine and ATP using a batch column chromatography according to the method described by Salomon [37]. We used Isoproterenol (Sigma) as positive control.

### Calcium Measurements in Live Cells

Cells were seeded into poly-D-lysine-coated 384-well plates (Cellcoat, Greiner bio-one) 24 h before the experiment at a concentration of 10000 cells per well in a total of 50 µl. On the day of the experiment, plates were flicked to remove the medium and cells were loaded with 40 µl of 1.6 µM FLUO-4-AM (Molecular Probes) in dilution buffer consisting of 20 mM HEPES (Gibco), 1x HBSS (Gibco) and 0.1% BSA (Calbiochem), supplemented with 2.5 mM Probenecid (Sigma) for 1 h at 37°C, 5% CO_2_. Cells were subsequently washed with 20 mM HEPES, HBSS, 2.5 mM Probenecid in a TECAN cell washer station and 30 µl of the buffer were left to cover the cells. Calcium-induced fluorescence was detected using the Functional Drug Screening System (FDSS7000, Hamamatsu). Baseline fluorescence was measured, followed by the pipeting of 15 µl of 3-times concentrated compound-containing solution onto the cells. Signals were taken 60-times every second and then 40-times every 2 seconds. The data were calculated as ΔF over F (maximum of response - baseline/baseline). For each assay, values were obtained in quadruplicates and data are expressed as mean +/− standard deviation. We used ATP (Tocris) as positive control.

### IP Formation Assay (PI Assay)

Confluent cells grown in 48 well plates were labeled with myo[^3^H]inositol (100 MBq/ml; ART/Anawa Trading) for 24 h in serum-free DMEM (Gibco) medium supplemented with 1% penicillin/streptomycin (Animed). The medium was removed and cells were washed once with HBSS buffer (Gibco). 90 µl of HBSS supplemented with 20 mM LiCl_2_ were added per well. Lithium blocks inositol monophosphatases activity, leading to the accumulation of inositol phosphates [38]. 90 µl of HBSS/20 mM LiCl_2_ (containing compounds where indicated) were then added per well. Cells were incubated for 20 min at 37°C. The stimulation was stopped and inositol phosphates were extracted with ice-cold formic acid. Total inositol phosphates were separated from free inositol using batch column chromatography and analyzed as described [39]. We used a positive control mix (each at 1 µM final) containing ATP (Tocris), LPA18∶1 (Cayman), S1P (Tocris), carbachol (Sigma) and bradykinin (Tocris).

### Immunofluorescence

A Cytofix/Cytoperm kit (BD Biosciences) was used for immunostaining. Cells were seeded into poly-D-lysine (Sigma)-coated LabtekII chambered coverglasses with cover (Nalge-Nunc) and cultured for 24 h at 37°C, 5% CO_2_. The liquid was subsequently removed and the coverglass air-dried. For immuno-staining, each well was washed once with 500 µl of PBS. Subsequently, 300 µl of Cytofix/Cytoperm fixation and permeabilization solution were added per well and cells were fixed for 20 min on ice. Cells were washed twice with 500 µl of Perm/Wash solution. 200 µl of blocking solution (1x Perm/Wash in 10 mM HEPES (Gibco), 140 mM NaCl, 6 mM CaCl_2_, 5% FBS, 1% BSA) were added and cells were incubated at least 15 min at room temperature at 4°C. The buffer was removed and 100 µl of blocking buffer containing the primary antibody (anti-V5; Invitrogen) at a dilution of 1∶500 was added to each well. The antibody solution was removed after 2 to 4 h of incubation at 4°C, and cells were washed twice with 500 µl of Perm/Wash solution. Subsequently, 100 µl blocking buffer containing the secondary antibody (Alexa Fluor 488-labeled chicken anti-mouse; Molecular Probes) at a dilution of 1∶500 were added for 1 h at 4°C. Again, cells were washed twice with 500 µl of Perm/Wash solution. For nuclear staining, 500 µl of DAPI solution (Roche, 1 mg/ml in water) were added at a 1∶1000 dilution in PBS for 2 to 3 min prior to a PBS wash. Finally, cells were covered with 400 µl of PBS and subjected to microscopic examination.

### Organoid Cultures

Organoid cultures from adult C57Bl/6 mice were established and maintained essentially as described [8]. In brief, cultures were embedded in Matrigel basement membrane (BD Biosciences) and cultivated in advanced DMEM/F12 (Invitrogen) containing gentamycin, penicillin-streptomycin, L-Glutamine (Invitrogen) and supplemented with 50 ng/ml Egf (Peprotech), 100 ng/ml Noggin (Peprotech) and 250 ng/ml recombinant human RSPO1 (purified from conditioned medium of transfected HEK293 cells). For CHIR99021 treatment, organoids were passaged as described [8] and freshly plated in the above medium without RSPO1 but in the presence of 5 uM CHIR99021 [40]. Timed matings were performed using heterozygous Lgr4 KO (Lgr4-mCherry-IRES-CreERT2) mice or Lgr5 KO (Lgr5-EGFP-IRES-CreERT2) mice, respectively (manuscript in preparation). Pregnant females were euthanized at embryonic day (E) 16.5 or E18.5 to obtain wild-type embryos as well as heterozygous and homozygous Lgr4 or Lgr5 KO embryos. Intestines from euthanized embryos or adult C57Bl/6 wild-type mice were dissected and organoid cultures were established and maintained essentially as cultures derived from adult mice.

### Statement on Animal Welfare

All experiments were carried out in accordance with authorization guidelines for the care and use of laboratory animals. Studies described in this report were performed according to Novartis animal license number 1022.

## Supporting Information

Figure S1 **Multiple siRNAs targeting LGR4 reduce RSPO1-induced Wnt pathway activation.** 4 siRNAs targeting LGR4 were designed and tested for their abilities to knock-down LGR4 mRNA levels in HEK293T cells. (a) RSPO1- but not Wnt3a-induced STF reporter activities are reduced by LGR4 siRNA’s 1 (4–1) and 2 (4–2) but not 3 (4–3) and 4 (4–4) compared to the pGL2 negative control. (b) Taqman qPCR data reveal that LGR4 siRNA’s 1 and 2 but not 3 and 4 reduced LGR4 mRNA levels. (c) Relative expression levels of LGR4, LGR5 and LGR6 in HEK293T cells as assessed by qPCR; Ct, Threshold cycle.
(EPS)

Figure S2 **The C-terminal Cys-rich domain within the LGR4 ECD is not important for RSPO1-induced Wnt activation.** (a) HEK293T cells were transiently co-transfected with pSTF, pRenilla and one of the following V5-tagged expression constructs, each: LacZ control (LacZ), full-length LGR4 (FL), and LGR4 harboring deletions aa. 465–533 (C-Cys-rich domain, ΔC-Cys) or 930–951 (C-terminus, ΔC-term). One day later, cells were treated with either 50 ng/ml RSPO1 or left untreated, and luminescence values were determined after 24 h of treatment. (b) The expression constructs used for assessing RSPO1 responsiveness were transiently transfected into HEK293T cells and analyzed for their relative expression levels by Western blot analysis. A V5 tag-specific antibody was used as primary antibody. The respective LGR4 deletion mutants are marked with black arrows. (c) The same expression constructs were transiently transfected into HEK293T cells and analyzed for their sub-cellular localization by immunofluorescence analysis using a V5 tag-specific primary antibody.
(EPS)

Figure S3 **hCG is not able to induce LGR2-mediated Wnt/β-catenin signaling.** (a) human choriogonadotropin (hCG), the ligand of LGR2, induced increased cAMP level in HEK293T cells overexpressing LGR2. (b) HEK293T-STF cells inducibly expressing LGR2 were treated with increasing concentrations of hCG in the presence or absence of 5% Wnt3a CM. luciferase values were determined 24 h following induction. No effect of hCG on Wnt reporter induction could be measured under any condition.
(EPS)
